# Multi-Genetic Marker Approach and Spatio-Temporal Analysis Suggest There Is a Single Panmictic Population of Swordfish *Xiphias gladius* in the Indian Ocean

**DOI:** 10.1371/journal.pone.0063558

**Published:** 2013-05-22

**Authors:** Delphine Muths, Sarah Le Couls, Hugues Evano, Peter Grewe, Jerome Bourjea

**Affiliations:** 1 Délégation Océan Indien, IFREMER, Le Port, France; 2 CSIRO Marine and Atmospheric Research, Hobart, Tasmania, Australia; Technical University of Denmark, Denmark

## Abstract

Genetic population structure of swordfish *Xiphias gladius* was examined based on 2231 individual samples, collected mainly between 2009 and 2010, among three major sampling areas within the Indian Ocean (IO; twelve distinct sites), Atlantic (two sites) and Pacific (one site) Oceans using analysis of nineteen microsatellite loci (n = 2146) and mitochondrial ND2 sequences (n = 2001) data. Sample collection was stratified in time and space in order to investigate the stability of the genetic structure observed with a special focus on the South West Indian Ocean. Significant AMOVA variance was observed for both markers indicating genetic population subdivision was present between oceans. Overall value of F-statistics for ND2 sequences confirmed that Atlantic and Indian Oceans swordfish represent two distinct genetic stocks. Indo-Pacific differentiation was also significant but lower than that observed between Atlantic and Indian Oceans. However, microsatellite F-statistics failed to reveal structure even at the inter-oceanic scale, indicating that resolving power of our microsatellite loci was insufficient for detecting population subdivision. At the scale of the Indian Ocean, results obtained from both markers are consistent with swordfish belonging to a single unique panmictic population. Analyses partitioned by sampling area, season, or sex also failed to identify any clear structure within this ocean. Such large spatial and temporal homogeneity of genetic structure, observed for such a large highly mobile pelagic species, suggests as satisfactory to consider swordfish as a single panmictic population in the Indian Ocean.

## Introduction

Large pelagic species have commonly been thought to lack genetic spatial structure due to their cosmopolitan distribution, large population size, high fecundity, production of numerous pelagic larvae and ability to easily migrate inter-ocean distances [1]. However, examples of single panmictic worldwide distributed populations appear to be rare such as in the case of the wahoo, *Acanthocybium solandri* [2]. Long-distance migrants are, by definition, highly mobile but factors maintaining observed genetic population structure are poorly understood yet these are common (examples such as Atlantic Bluefin tuna [3], bigeye tuna [4]). Structure has often been observed for large pelagic species with cosmopolitan distribution demonstrating genetic population structure partitioned among or even within ocean basins. Examples of genetic sub-structure in cosmopolitan species include albacore tuna *Thunnus alalunga* [5] and at the intra-oceanic level, the blue marlin *Makaira nigricans* [6] or the white marlin *Tetrapturus albidus* [7]. Geographic partition was also shown in the most widely distributed species of pelagic fishes, the broadbill swordfish *Xiphias gladius* with two subdivisions in the Pacific Ocean (North-west *versus* South-East; [8]), as well as in the Atlantic Ocean (North-west *versus* South [9]), with a Mediterranean population clearly isolated from those of the Atlantic Ocean [10]. Furthermore, evidence of northwest and south Atlantic stocks of swordfish was supported by both parasite [11] and genetic data [9] strongly demonstrating that this species has the potential to evolve intra-ocean population sub-division.

Genetic divergence in migratory species may exist if individuals are philopatric and consistently return to the same breeding grounds. Conventional and electronic tagging of swordfish shows that individuals are capable of extensive movements of thousands of kilometres [12] across the North Atlantic - 2500 km [13], [14] and even larger migration in the Indian Ocean with one swordfish recaptured 6670 km south-eastward from the point of release (975 releases –29 recaptures over the last 20 years; [15]). However, migratory behavior of swordfish seems to be constrained and delineated by equatorial boundaries, at least in the Atlantic Ocean [12], explaining why genetic pattern was more similar between southern regions of two adjacent oceans than from the southern to the northern parts of a same ocean [16], [17]. Moreover, a great number of tagged swordfish were recaptured near the release site [13], [18], [19]. For instance, in the North Atlantic, Neilson et al. [14] showed evidence of precise homing from nesting to feeding areas on 4 swordfishes over 25 tracked individuals. This homing behaviour thus may explain discrete mtDNA boundaries observed between samples from North-west Atlantic and South Atlantic known to be distinct breeding grounds [20].

Swordfish population structure has previously been explored within the Indian Ocean with most studies identifying some genetic differentiation in the Indian Ocean [17], [21], [22], [23], although there appears to be inconsistency among the various conclusions. However, these studies were in fact conducted on different geographic scales, with too few individuals analyzed, the reproduction behavior was not taken in consideration, there were heterogeneous sampling periods, and often only one genetic marker was used. This could explain incongruency of results among studies in terms of stock structure. A hypothesis of strong philopatry would suggest that genetic differentiation could be observed in the Indian Ocean between different spawning grounds and/or between Northern and Southern hemispheres like in the other oceans. However, reproduction data are scarce in the Indian Ocean (see review in: [24]) and reproductive season and spawning ground remain unclear. Three spawning grounds were however described: the Gulf of Bengal and off the Somalia coast - where spawning is supposed to occur after April for both areas - and around Reunion island - where spawning is supposed to take place from October to April. Evidences from past studies suggest that spawning may occur in the West Equatorial area [25] the Gulf of Bengal [26] and the East Madagascar Tropical area from October to April [24]. To date no genetic study has investigated population structuring of Indian Ocean swordfish with a sufficient sampling strategy to address population structure within the Indian Ocean.

The identification of genetic structure in the Indian swordfish should be of importance in term of fish management as one of the challenging issues commonly recognized is to match the artificial spatial scale of stock assessment with the natural spatial structure of the species [27]. Swordfish has the largest commercial value among billfish fisheries and is currently heavily exploited by commercial fisheries in the Indian Ocean. On the basis of the last swordfish stock assessment [28], levels of catches in the whole Indian Ocean for 2006–2010 (average of 24 008 tons) were considered below the estimated *maximum sustainable yield (*MSY; 29 900–34 200 tons). Nevertheless, when population structure was considered and when the assessment focused on the southwest Indian Ocean as an independent stock – a case considered by the India Ocean Tuna Commission (IOTC) on the basis of the fishery data [29], most of the evidence indicated that the resource has been overfished in the past decade, with the current level of catches indicating a stock fully exploited (8 112 tons in 2010 with an estimated MSY: 7 100–9 400; [26]). Therefore, deeper investigation on the swordfish stock structure was recommended as one of the top priorities by the IOTC Scientific Committee to reduce the uncertainty in assessment [30].

The present study aims to determine the swordfish genetic population structure in the Indian Ocean. For this purpose, an intensive sampling was conducted over the whole Indian Ocean, at several periods during two consecutive years (2009–2010). We examined genetic variation of more than two thousands swordfish using newly developed genetic markers, supposedly more discriminating than older ones: 19 microsatellite loci [31], [32] and mitochondrial sequences of the Nicotinamine Dehydrogenase subunit 2 (ND2) [22].

## Materials and Methods

### Sample Collection

**Figure 1:**
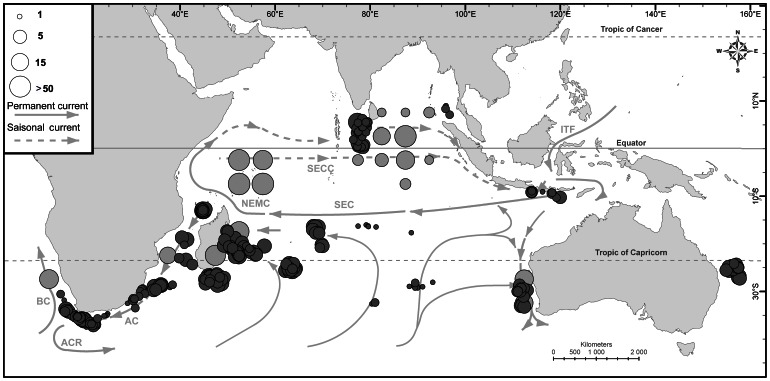
Geographic location of Swordfish tissue samples analysed in this study. The size of circles is proportional to number of swordfish sampled; the colour indicates the accuracy of the localisation data collected (dark grey for exact coordinates and light grey for 5° square position). This map also shows the main Indian Ocean currents (from Schott et la. 2009); SEC: South Equatorial Current; SECC: South Equatorial Counter Current; NEMC: Northeast Madagascar Current; AC: Agulhas Current; ACR: Agulhas Current Retroflexion; BC = Benguela Current; ITF: Indonesian Trough Flow.

Sampling for the present study focused on the Indian Ocean (IO) – as defined by international conventions as the waters delineated from the Atlantic Ocean by the 20° east meridian, from the Pacific by the meridian of 146°55′ east and a southern limit at 60°S [33]. Ethical approval was not required for this study, as all fish were collected as part of routine fishing procedures. Swordfish samples were collected from different zones within the Indian and adjacent Oceans (Figure 1, Table 1 and Appendix F1 in File S1) by onboard observers on commercial fishing vessels or at landing (with due care collecting the related fishing information). Swordfish were killed by the fishermen by cutting their head off. For each sample, muscle tissue biopsies were so taken on already dead animals; they were then stored in 90% ethanol then frozen until DNA was isolated. Information on sample location (exact latitude and longitude or 5° square) was systematically noted. Whenever it was possible fish sex information were collected.

**Table 1:** Swordfish sampling and genetic characteristics from the Indian and adjacent Oceans sampling areas.

Sampling							ND2 sequences							Microsatellite			
Ocean	Sample set name	Geographic area	Dates		Corres-ponding season	Sex ratio	N	Nhap	*h*	π	Main Hap. #11	Private hap.	Fu’s F	N	Rs	Fis19	Fis16
			(begin	- end)						‰		%					
**INDIAN OCEAN**	**AUS_08_1**	Australia (West)	22/02/08	02/03/08	–	NA	36	16	0.866	2.202	0.33	0	**−9.5**	38	6.4	**0.088**	**0.036**
	**AUS_08_2**	Australia (West)	25/11/08	30/11/08	–	NA	27	16	0.866	1.573	0.37	0	**−15.5**	28	6.3	**0.108**	0.048
	**AUS_09_1**	Australia (West)	12/12/09	15/12/09	2	NA	27	14	0.803	1.954	0.44	0	**−9.3**	29	6.0	**0.110**	0.045
	**AUS_11_1**	Australia (West)	28/05/11	31/05/11	–	NA	17	9	0.860	1.544	0.35	0	**−5.1**	19	5.8	**0.107**	**0.060**
	**CA1_10_1**	Mozambic chanel (North)	06/04/10	17/04/10	4	0.55	42	26	0.914	2.213	0.28	0	**−26.7**	47	6.1	**0.092**	0.030
	**CA2_10_1**	Mozambic chanel (South)	03/01/10	04/02/10	3	NA	53	29	0.912	2.735	0.28	0.05	**−26.1**	53	6.4	**0.108**	**0.067**
	**GB1_09_1b**	Gulf of Bengal (West)	27/09/09	09/12/09	2	NA	40	23	0.890	2.097	0.32	0.02	**−22.9**	48	5.9	**0.107**	**0.046**
	**GB1_10_1**	Gulf of Bengal (West)	27/06/10	30/07/10	4	NA	77	43	0.927	2.231	0.25	0	**−27.1**	83	6.1	**0.100**	**0.030**
	**GB2_10_1**	Gulf of Bengal (central)	21/01/10	21/01/10	3	NA	31	19	0.944	2.361	0.19	0.03	**−15.8**	38	5.8	**0.064**	0.010
	**GB2_10_2**	Gulf of Bengal (central)	12/12/10	22/01/11	5	NA	19	11	0.888	1.731	0.31	0	**−7.2**	21	6.0	**0.061**	**0.052**
	**IND_09_1**	Indonesia	02/05/09	12/06/09	1	NA	41	22	0.891	1.995	0.31	0.07	**−21.1**	40	6.2	**0.128**	**0.055**
	**MAD_09_1**	Madagascar (South-east)	28/04/09	10/05/09	1	0.74	42	21	0.890	2.918	0.30	0.07	**−13.4**	91	6.2	**0.080**	0.020
	**MAD_09_2**	Madagascar (South-east)	07/11/09	18/11/09	2	0.74	93	45	0.838	1.945	0.39	0.05	**−27.4**	97	6.1	**0.116**	**0.053**
	**MAD_10_1**	Madagascar (South-east)	21/04/10	01/05/10	4	0.70	93	40	0.908	3.456	0.27	0.11	**−26.1**	94	5.8	**0.060**	0.022
	**MAY_09_1**	Mayotte	26/10/09	11/12/09	2	NA	85	39	0.881	2.210	0.32	0.10	**−27.1**	95	6.2	**0.103**	**0.053**
	**MAY_10_1**	Mayotte	12/10/10	18/11/10	5	NA	76	30	0.892	2.149	0.30	0.03	**−27.0**	68	5.8	**0.064**	**0.037**
	**RON_09_1**	Rodrigues (North)	26/09/09	06/10/09	2	0.66	70	39	0.906	2.730	0.30	0.08	**−26.6**	81	6.0	**0.143**	**0.088**
	**ROS_09_1**	Rodrigues (South)	09/10/09	18/10/09	2	NA	11	8	0.927	2.109	0.27	0	**−4.2**	15	5.9	**0.116**	**0.085**
	**ROS_10_1**	Rodrigues (South)	19/05/10	24/05/10	4	0.70	34	20	0.921	2.046	0.26	0.05	**−18.6**	35	6.2	**0.051**	**−**0.008
	**ROS_10_2**	Rodrigues (South)	20/07/10	26/07/10	4	0.71	44	23	0.887	2.001	0.31	0.13	**−22.3**	45	6.1	**0.049**	0.001
	**ROS_10_3**	Rodrigues (South)	12/10/10	18/10/10	5	0.67	84	35	0.801	2.177	0.44	0.08	**−27.1**	78	6.2	**0.033**	**−**0.007
	**RUN_09_1**	Reunion island	29/05/09	03/06/09	1	0.5	63	26	0.795	1.746	0.44	0.07	**−26.4**	63	6.0	**0.123**	**0.051**
	**RUN_09_2**	Reunion island	15/10/09	29/11/09	2	0.67	59	27	0.916	2.891	0.23	0.05	**−20.3**	73	5.9	**0.108**	0.031
	**RUN_10_1**	Reunion island	16/06/10	31/07/10	4	0.56	78	39	0.921	2.695	0.25	0.08	**−26.6**	93	6.1	**0.046**	0.015
	**RUN_10_2**	Reunion island	21/10/10	22/11/10	5	0.47	92	41	0.903	1.999	0.28	0.08	**−27.4**	85	6.1	**0.069**	**0.028**
	**SEY_09_1**	Seychelles	22/11/09	17/12/09	2	0.93	85	34	0.826	2.017	0.40	0	**−27.3**	91	6.1	**0.092**	0.027
	**SEY_10_1**	Seychelles	02/07/10	08/07/10	4	NA	67	28	0.874	1.888	0.32	0	**−27.2**	68	6.2	**0.109**	**0.048**
	**SEY_10_2**	Seychelles	04/11/10	17/11/10	5	NA	21	11	0.814	1.581	0.42	0	**−7.2**	21	6.2	**0.087**	0.046
	**SEY_11_1**	Seychelles	21/01/11	21/01/11	5	NA	24	15	0.837	2.228	0.41	0	**−11.0**	30	6.3	**0.092**	0.029
**ATLANTIC OCEAN**	**AFS_09_1b**	South Africa	29/07/09	01/11/09	2	NA	64	32	0.929	3.064	0.23	0.09	**−26.1**	53	6.4	**0.075**	**0.036**
	**AFS_10_1**	South Africa	24/01/10	04/02/10	3	NA	15	11	0.904	2.857	0.33	0	**−6.0**	15	6.2	**0.021**	0.008
	**AFS_10_2**	South Africa	21/04/10	26/04/10	4	NA	49	30	0.916	2.493	0.28	0	**−26.7**	49	6.2	**0.086**	0.027
	**AFS_10_3**	South Africa	25/10/10	30/10/10	5	0.70	10	9	0.977	2.911	0	0	**−5.6**	12	5.8	**0.117**	0.080
	**NAM_11_1**	Namibia	10/11		–	NA	24	13	0.873	1.996	0.16	0	**−8.2**	23	6.5	**0.023**	**−**0.026
**PACIFIC OCEAN**	**COR_09_1b**	Coral Sea	10/04/09	08/07/09	1	NA	50	20	0.884	2.076	0.30	0.10	**−14.4**	53	6.5	**0.061**	0.002
	**COR_09_3**	Coral Sea	26/10/09	08/11/09	2	NA	63	33	0.916	2.229	0.26	0.15	**−27.0**	72	6.2	**0.047**	0.009
	**Out Class**		04/07/08	27/02/11	–	0.92	185	65						202			
	**TOTAL**					0.67	2001	282						2146			

- Sampling characteristics: GPS coordinates, sampling dates and corresponding season, sex ratio (estimated as the number of females on the whole number of swordfis). Sampling sets (*i.e.* swordfish sampled in a given area at a given time) were named as XXX_00_## (XXX for the area name, 00 for the sampling year, ## for the period within the respective 00 year). ‘Out Class’ samples correspond to swordfish collected out of a sampling season and/or out of a sampling site.

- ND2 characteristics: *Nhap* the number of haplotypes per population, *h* the haplotype diversity, π the nucleotide diversity, the percentage of main ***(#11)*** and private haplotypes, Fu’s Fs value.

- Microsatellite characteristics: Rs the allelic richness (estimated on 10 individuals), *Fis19* and and *Fis16* the fixation index respectively estimated with 19 and 16 loci.

Significance at p<0.001 is noted by bold characters.

Initial sampling strategy was to sample one hundred fish per zone at two targeted seasons (April–June that we considered as the non-spawning season for this species and October–December, the season where swordfish should be in spawning condition) over two consecutive years (2009 and 2010). Initial sampling seasons were defined based on known information of swordfish reproductive condition in IO (see Introduction and [24], [25], [26]). Due to field realities, not all samples were collected during those periods but were in fact collected over 46 months from February 2008 to October 2011 (see Appendix F2 in File S1) and then classified in five seasons numbered from 1 to 5 (see Table 1).

### Genetic Analysis

Total genomic DNA was extracted using DNAeasy Tissue Kit (Qiagen) following the manufacturer instructions. A 1007 bp fragment of the mitochondrial ND2 gene was amplified by PCR using the primers and recommended conditions defined in Bradman et al. [22]. PCR products were purified and sequenced in forward and reverse directions on an ABI 3100 sequencer (Macrogen Inc.). Sequences were edited using Chromas version 1.6 [34] and aligned using CLUSTALW [35] in BIOEDIT Sequence Alignment Editor [36]. Sequences were submitted to GenBank (Accession numbers JQ353203 to JQ353484).

Nineteen microsatellite loci were also used, three from [32] (Xg-66, Xg-144, Xg-166) and sixteen from Bradman et al. [31]: A3, A4, A7, A8, A10, A113, A115, B108, B112, B6, C10, C4, C7, C8, D11, D2B. Reactions were performed in 20 µl containing 1X PCR buffer, 2.5 mM MgCl_2_, 2 µM of each dNTPs, 0.3 µM of each primer, 0.5 U of SilverStar Polymerase Taq (Eurogentec), 25 ng of genomic DNA. Cycling parameters were 93°C for 3 min, followed by 35 cycles of 93°C for 30 s, 50–62°C for 50 s, and 72°C for 50 s and a final elongation at 72°C for 30 min. PCR were amplified separately and electrophoresed in three multiplex panels. Amplified fragments were separated on an ABI Prism 3100 genetic analyser. Alleles were scored using a co-migrating size standard (Genescan500, Applied Biosystems, Inc.) and identified using GENE MAPPER v4 (Applied Biosystems Inc.).

### Data Analyses

Data analysis was first conducted on the whole dataset to identify global level of structure. We also defined spatially and temporarily stratified sampling sets (*i.e.* swordfish sampled in a given area at a given time; each sample set was named as XXX_00_## (XXX for the area name, 00 for the sampling year, ## for the period within the respective 00 year - see details in Table 1) for more meaningful comparisons (*e.g.* comparison of fish from a same area at different times or from different areas at a same time).

For ND2 sequences, haplotype (*h*) and nucleotide (π) diversities and Fu’s [37] F-statistic were estimated per sampling sets with DNAsp 5.0 [38]. Fu’s F-statistic tests for departure from equilibrium between the addition of variation by mutation and the removal of variation by genetic drift; theoretically, mutation-drift equilibrium should be reached if the effective population size has remained stable in the past. Phylogenetic relation between all available ND2 sequences were represented by a neighbour-joining tree constructed using MEGA 5 [39]. Correlations between haplotype frequencies and longitude were tested using Pearson coefficient. For microsatellites, allele frequencies, mean number of alleles (*Nall*), and the observed (*Ho*) and expected (*He*) heterozygosities [40] were calculated per samples sets with ARLEQUIN 3.5 [41]. To account for differences in sample size, allelic diversity was adjusted by estimating the allelic richness (*Rs*) using the rarefaction process of the standArich package (available at http://www.ccmar.ualg.pt/maree/software.php?soft=sarich) for R [42]. Deviations from Hardy-Weinberg equilibrium were examined for each sampling set, at each locus, by calculating Wright’s [43] fixation index *F_is_* as estimated by Weir and Cockerham [44] and tested using exact tests performed with Arlequin 3.5 [41]. MICRO-CHECKER 2.2.3 [45] was used to detect possible null alleles. Microsatellite dataset was analysed using the software STRUCTURE 2.3.2 [46] to determine if the genotypes could be partitioned in one or more genetic pools. For this analysis, an admixture model assuming independent allele frequencies was used and ten replicates were run (each with 1.10^5^ burn-in samples/generations and 5.10^5^ iterations) for *K* values from 1 to 5.

For both ND2 sequence and microsatellite data, the analysis package ARLEQUIN 3.5 [41] was used to estimate pairwise values of genetic differentiation. A total of 10 000 permutations were used with the fixation index Ф_st_ for sequence data and Wright’s F_ST_ statistic for microsatellite data. In both cases, critical significance levels for multiple testing were corrected in agreement with Narum [47] using a sequential Benjamini-Yekutieli procedure [48]. Pairwise values of genetic differentiation between sample sets were used as input data in order to construct neighbour-joining trees with the program MEGA 5 [39]. Jost’s [49] unbiased estimator of divergence (D, based on the effective number of alleles rather than on the expected levels of heterozygosity) was also calculated per pair of localities using the software SPADE (available at http://chao.stat.nthu.edu.tw/softwareCE.html) for ND2 sequences and SMOGD [50] for microsatellite data. To test for patterns of isolation-by-distance, marine distances between localities (estimated on the http://www.geodistance.com website) were plotted against genetic distance (using Ф_st_/(1− Ф_st_) for mitochondrial data or F_ST_/(1− F_ST_) for microsatellite data following the recommendations of Rousset [51]. The significance of this relationship was tested with a Mantel test, performed in R [42] using the *ncf* package (available at http://onb.ent.psu.edu/onb1/R).

ARLEQUIN 3.5 [41] was also used to perform analysis of molecular variance (AMOVA) with *a priori* grouping based on geographical or temporal proximity, within or between oceans. The software SAMOVA 1.2 [52] was finally used to perform spatial analysis of molecular variance (SAMOVA) on localities that were sampled within the same period. This approach could detect genetic barriers in a sampling region without *a priori* group definition and identify geographic partitions that maximize genetic differences between groups and geographic homogeneity within groups; it was tested for *K* group values ranging from 1 to 4, with 100 annealing replicates each time.

Finally, the same analytical approaches (pairwise values of genetic differentiation and AMOVA) were processed to assess whether or not sex of individual had an effect on the genetic structure.

## Results

A total of 2 231 swordfish were sampled from the three major study areas during this study (1920 from the IO; 186 from Atlantic Ocean; and 125 from the Pacific Ocean). Sampling details are provided in Table 1. A total of 2 146 were genotyped with microsatellites and 2 001 were sequenced at the mitochondrial ND2 gene.

### Genetic Diversity

#### ND2 sequences

A total of 195 variable sites, constituting 282 haplotypes, were detected among the ND2 sequences (1007 bp) of 2 001 swordfish. Approximately 48% of these 282 haplotypes were represented more than once. Mean haplotype diversity (H_d_) and mean nucleotide diversity (π) were high, respectively 0.886 (±0.04) and 0.0022 (±0.0004), and similarly high within each sample set (Table 1). The most common haplotype (#11) was well represented in all localities, except AFS_10_3, where it was absent. The mean frequency of this haplotype per sampling locality was 31% (±7%), varying from 16% in NAM_11_1 to 44% in AUS_09_1, ROS_10_3 and RUN_09_1 (Table 1). The private haplotypes constituted a small proportion of the individuals, with a mean frequency of 4% per sample set and the highest frequency of 13% in ROS_10_2 (Table 1). Fu’s F values were highly negative and significant (F = −682.3, p<0.001; Table 1) and the mismatch distribution for overall dataset presented one peak, implying that the sudden population expansion model could not be rejected (Appendix F3 in File S1).

**Figure 2:**
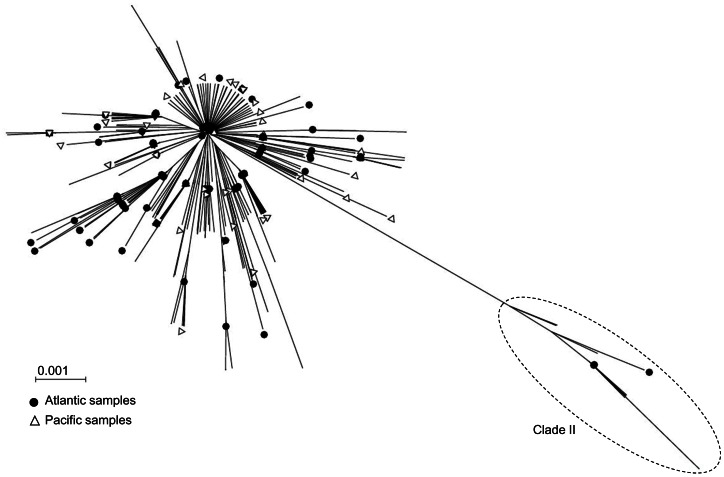
Unrooted neighbor-joining tree showing the relationship between the ND2 sequences (n = 2001). White triangles are samples from the Pacific Ocean (5.5% of the samples), black circles from Atlantic Ocean (8% of the samples) and branches without symbol are from Indian Ocean (86.5% of the samples). Clade I and clade II names refer to the same nomenclature proposed in Alvarado-Bremer *et al.*, (2005a).

The relationship between ND2 sequences as represented by a neighbor-joining tree is shown in Figure 2. Sequence analysis revealed two divergent clades, a dominant one, which contained 98% of the samples, and a second clade separated by seven fixed mutations from the main clade. This clade structure observed in this study was similar to one previously described from examination of mitochondrial cytochrome b sequence data [53]. By analogy to the aforementioned study, the clades herein were called clade I and clade II respectively for the common and the rare clades. The clade I is equally represented in all sample sets while the clade II is absent from the Pacific Ocean sample sets (COR_09_1 and COR_09_3). The proportion of the clade II within each sample was thus significantly and negatively correlated to decreasing longitude (r = 0.14; p<0.02).

#### Microsatellites

Allelic richness was of the same order between the different sample sets, with a mean at 6.16 (±0.19) varying from 5.8 for MAD_10_1 to 6.50 for COR_09_1 (Table 1). Fixation indices Fis were highly significant in most sample sets with values ranging from 0.02 to 0.14 (Table 1), mostly because of significant heterozygote deficiencies at the three loci A3, B6 and B108. These loci were indeed characterised by the presence of null alleles (p<0.05); consequently, these three loci were excluded from following analyses which were were therefore run using 16 loci. Fis values became non-significant for more than half of the sample sets when removing these 3 loci (Table 1). No loci were in disequilibrium (p<0.001) over the whole dataset, supporting the independent assortment of alleles at different loci.

### Inter-ocean Structure

#### ND2 sequences

**Figure 3:**
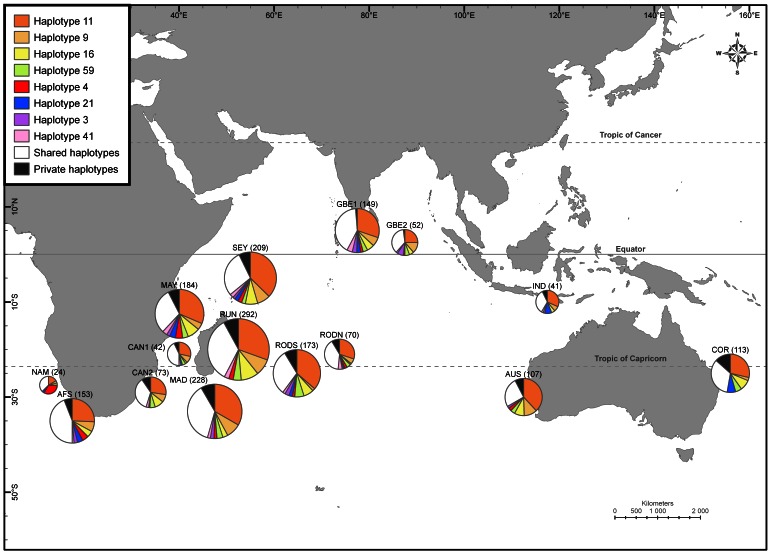
Map of plotted frequencies of the main ND2 haplotypes, shared haplotype and private haplotypes per identified areas. Number of samples is shown in brackets.

Overall Ф_ST_ was 0.006 (p<0.001) when considering all the samples; it decreased to 0.001 and was non-significant (p>0.05) when considering only the swordfish sampled within the IO (i.e. excluding all the sample sets from NAM, AFS and COR areas). Pairwise genetic distance estimates (Ф_ST_) between sample sets are summarized by a neighbor-joining (NJ) tree in Appendix F4 in File S1. This analysis clearly segregated NAM_11_1 and AFS_09_1 from all the others sets but showed no clear structure among the Indo-Pacific sample sets. Of the 630 pairwise comparisons used in this NJ tree (see complete table in Appendix S1), 80 were significant (p<0.05) from which 64 concerned interoceanic comparisons (i.e. including at least one sample set from NAM, AFS or COR areas in the pairwise comparisons). The highest Ф_ST_ values were observed for the NAM sample sets (mean Ф_ST = _0.098), followed by COR and AFS comparisons (0.012 and 0.010 respectively). Consistent with these significant differentiation results, values of Jost’s D were very high when comparisons included NAM samples (D >0.25), even if the highest values of Jost’s D regarded AFS_10_3 (mean D = 0.74). An AMOVA analysis undertaken with grouping made per ocean also demonstrated a small but significant level of structure between oceans (Φ_CT_ = 0.011, p<0.001; see Table 2). This inter-ocean differentiation could be partly explained by the geographical distribution of haplotypes (shown on Figure 3). First, all areas except NAM (Atlantic Ocean) were dominated by the most common haplotype (#11) while NAM area was dominated by a secondary haplotype (#4; 33.3%), present in most areas but in a lower proportion. The proportion of the haplotype #4 in the AFS sample sets was highly variable according to sampling sets (from 0% in AFS_10_1 to 10% in AFS_10_3 with intermediate values of 2% in AFS_10_2 and 8% in AFS_09_1). Then, the COR area (Pacific Ocean) showed an absence of the haplotypes #4 and #41 and a higher proportion of haplotype #21 (Figure 3). The IND_09_01 sample set (Indonesia, the closest site to Pacific Ocean) was characterized by an absence of haplotype #4 only one haplotype #41 while the haplotype #21 is the most dominant one (10%) after haplotype #11.

**Table 2:** Results of AMOVAs made for both markers according to different grouping (bold underline significant values).

		ND2				Microsatellite			
Source of variation		d.f.	% variation	Fixation index	p	d.f.	% variation	Fixation index	p
**(1)**	Among oceans (Φ_CT_)	2	1.18	0.0118	**<0.001**	2	0.09	0.0008	**0.01**
	Among areas within oceans (Φ_SC_)	33	0.30	0.0030	0.02	33	0.26	0.0026	**<0.001**
	Among individuals within areas (Φ_ST_)	1770	98.52	0.0118	**<0.001**	3852	99.65	0.0035	**<0.001**
**(2)**	Among 3 groups within IO only (Φ_CT_)	2	0.08	0.0008	0.21	2	0.03	0.0003	0.09
	Among areas (Φ_SC_)	26	0.12	0.0012	0.12	26	0.25	0.0025	**<0.001**
	Among individuals within areas (Φ_ST_)	1502	99.80	0.0020	0.09	3305	99.72	0.0028	**<0.001**
**(3)**	Among 5 seasons within IO (Φ_CT_)	3	−0.14	−0.0010	0.98	4	0.02	0.0002	0.14
	Among areas within seasons (Φ_SC_)	22	0.34	0.0019	<0.05	21	0.25	0.0025	**<0.001**
	Among individuals within pop. (Φ_ST_)	1435	99.8	0.0033	<0.06	3138	99.73	0.0027	**<0.001**

(1) between the 3 oceans.

(2) among 3 geographical groups within IO, among SEIO (Australia and Indonesia), Gulf of Bengal and SWIO (all the others).

(3) among the 5 seasons within the IO (see Table 1 for details).

#### Microsatellites

The Structure analysis suggested that the highest likelihood of obtaining such data was to consider that only one genetic pool existed (K = 1). The likelihood decreases when estimates were made with more than one pool (over ten independent simulations: LnP(D) for K = 1 and K = 2 were −109850 and −110440, respectively). When considering two genetic pools, the mean posterior probability per individual is 0.50 (±0.04) providing more evidence against subdivision.

Overall F_ST_ was 0.0028 (p<0.001) when considering all the sample sets and 0.0026 (p<0.001) when considering only the swordfish sampled within the IO suggesting the same low level of structure within and between oceans. The neighbor-joining tree based on the pairwise F_ST_ estimates (Appendix F4b in File S1) failed to reveal any clear structure within the dataset. Of the 630 pairwise comparisons used in this NJ tree (see complete table in Appendix S1), 96 were significant (p = 0.000) from which 39 concerned interoceanic comparisons (i.e. including at least one sample set from NAM, AFS or COR areas). Consistent with these lack of clear structure, values of Jost’s D were very low (all D <0.03) even between oceans. However, an AMOVA analysis made with grouping done per ocean demonstrated a significant F_CT_ value (0.0008, p<0.01; see Table 2), as for ND2 sequences. The mean F_ST_ values for the NAM, COR and AFS comparisons were higher than for intra-ocean comparisons (0.006, 0.004 and 0.004 respectively while it was 0.003 among the Indian Ocean sample sets).

### Within Indian Ocean Analysis

We considered only the IO sample sets, excluding the five ones from the Atlantic Ocean (NAM and AFS) and the two from Pacific Ocean (COR). Irrespective of which marker was examined, the AMOVA analysis conducted within the IO showed that more than 99% of the variance was observed within the samples with no variance significantly associated with the partition into any kind of grouping (Φ_CT_ and F_CT_ <0.001, p>0.05; see Table 2). There is no clear partitioning between sample sets from a same site within the same season or between different sites within a same season. Similarly, the SAMOVA analysis failed to demonstrate any population subdivision using any of the two markers (less than 1% of genetic variance, p>0.05); without any *a priori* geographic grouping, between-group variance was maximized when one sample set was considered isolated from all the others.

**Figure 4:**
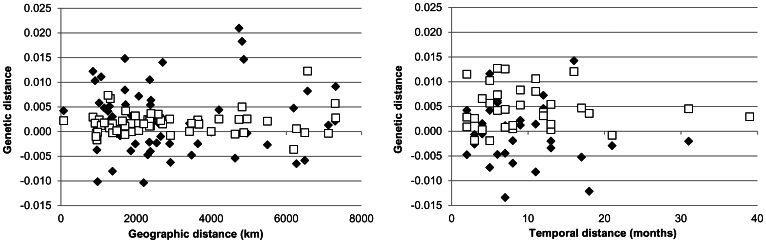
Isolation-by-distance and Isolation-by-time graphs. Graphs showed corrected pairwise genetic distances [Фst/(1−Фst) for mtDNA and Fst/(1−Fst) for microsatellite] plotted as a function of geographic distances (**4a**) or of time (**4b**) for Indian Ocean swordfish. Black diamonds are for mtDNA data and white squares for microsatellite data (all were not significant: Mantel tests with p>0.05).

Most ND2 pairwise values of differentiation (Appendix S1) were low and not significant, even between the most distant areas (*e.g.* AUS_08_1 versus RUN_10_1, Φ_ST_ = **−**0.0064, p>0.05). No significant differences were observed between years or seasons sampled at any site (*e.g.* AUS_08_1, AUS_08_2, AUS_09_1 and AUS_11_1; p>0.05*)* neither there was significant differences observed between different sites within a season (*e.g*. IND_09_1, MAD_09_1 ROS_09_1, MAY_09_1 and RUN_09_1; p>0.05). Looking at microsatellite pairwise F_ST_ values (Appendix S2), 139 upon the 240 comparisons appeared significant (p<0.05; 61 even highly significant, p<0.000). However, values of Jost’s D were very low with only 19 values above D = 0.01 (Appendix S2) at the intra IO level and no clear pattern of structure could be detected (Appendix F4b in File S1). For both sets of markers, there was no general trend for higher genetic divergence with increasing geographic or time separation (Figure 4). In other words, no isolation-by-distance nor isolation-by-time pattern were identified (all r <0.23 and all p>0.05).

Sex and maturity stage information were only available for localities in the South West Indian Ocean region (see Table 1). Most of the swordfish sexed were in stage maturity 1–2, i.e. 73% were immature individuals; analysis based only on mature swordfish could only be based on 114 individuals of the SWIO thus they were not done. Genetic structure partitioning was investigated by sex for sample sets where information was available (see Table 1; only in the South West Indian Ocean region). Overall Ф_ST_ was 0.0018 (not significant; p>0.05) when considering all the samples from this area; it was still not significant and decreased to 0.0009 (p>0.05) when considering only females and increased to 0.0020 (p>0.05) when considering males. A similar trend but significant was observed with microsatellite data as overall F_ST_ was 0.0023 (p<0.001) when considering all the samples, decreased to 0.0010 (p<0.05) when considering only the females and increased to 0.0040 (p<0.005) when considering the males. This trend indicated a higher genetic homogeneity between females than between males at the scale of the SWIO.

## Discussion

### Homogeneity within the Indian Ocean

This study aimed as a first step to evaluate genetic structure of the swordfish *Xiphias gladius* within the Indian Ocean (IO). The results obtained from both ND2 sequences and microsatellites on multiple sampling areas over multiple seasons detected some level of genetic heterogeneity but failed to demonstrate evidence that swordfish have multiple discrete populations within the IO. All the analysis focusing on the important spatially and temporally stratified sampling done in the SWIO failed to identify any significant structure. These results are consistent with the existence of a single panmictic population in the Indian Ocean population and thus they contradict previous studies suggesting the presence of at least two potential genetic populations [16], [17], [23]. Previous studies were in fact conducted on small geographic areas, with few individuals opportunistically collected and often only one genetic marker was used, leading to a potential artifact genetic subdivision. Present results are more consistent with main swordfish life history traits, *i.e.* long distance migration observed for IO swordfish using tag-recapture approach [15], [54] and fecundities of several millions of eggs per female [55]). The significant negative Fu’s values and the unimodal mismatch analysis could be interpreted as a pattern of recent demographic expansion. Together with the very high levels of genetic diversity and the lack of differentiation detected at a large spatial scale, this might be well in agreement with the assumption of a large population size and long range dispersal typical of large pelagic fishes.

The existence of at least two distinct stocks observed for this species in the Atlantic and Pacific Oceans [8], [20] could be partly explained by the existence of cold and productive waters in both North and South extremes of these oceans which are divided by warm oxygen poor waters of the equatorial region separating these two areas; admixture in same feeding areas of population breeding in different hemisphere was thus limited. Similarly, the IO is also divided into two hemispheres by the Equator, involving the known bio-ecological consequences in the marine realm driven by latitudes. This Ocean is also globally characterized by the westward South Equatorial Current (SEC; [56]) and around 12°S by the hydrochemical South Tropical Front which separated two large oligotrophic areas, the Indian Monsoon Gyre Province (*MONS)* in the north and the Indian South Subtropical Gyre Province (*ISSG*) [57] (see Appendix F1 in File S1). However, none of these physical and ecological geographic separations seem to impact on the observed genetic structure of the swordfish as they appear to do in the Atlantic and Pacific Oceans. One main difference between the IO and the two other oceans is that the first one could be defined as ‘closed’ by continent in the north, with consequently less latitudinal variation and most importantly no cold water in the north. However, the Arabian Large Marine Ecosystem (*ALME* - which bordered the north west of the *MONS* province; Appendix F1 in File S1) is one of the most intense large scale seasonal coastal upwelling [58], thus considered as a highly productive ecosystem [59] and even as one of the most important phytoplanktonic bloom systems in the world [60], [61]. Such a specific oceanographic pattern makes *ALME* a serious candidate for a discrete feeding area; unfortunately, our sampling scheme did not allow us to identify whether this northwestern area is a specific foraging ground for some IO swordfish. Further exploration into the foraging behaviour of the swordfish, a deep-dwelling predator, with specialization on prey sizes and species [62], [63], [64], could be one of the key elements to explain the lack of genetic structure presently observed in the IO.

Genetic analysis made by sex also failed to reveal any structure, however there was an indication of a higher genetic homogeneity between females than between males at the scale of the SWIO (significant using the nuclear marker but not using the mtDNA one). The fact that the genetic information given by the two genders is not the same could indicate a sex-biased dispersal in which gene flow between populations is accomplished primarily by one gender [14], [65]. In the present case, one could speculate there was a higher dispersal for females than males. However, this contradicts results from both a previous study undertaken in the SWIO which showed more pronounced homing behavior in females [23], and with the common pattern of higher dispersal abilities recognized for male swordfish [66]. The discrepancy in conclusions between these two SWIO studies, associated with the low level of structure observed and the unclear genetic structure found using microsatellites may thus be interpreted as an indication of a lack of structure within the SWIO and *in extenso* is another argument suggesting a homogeneous single panmictic population in the IO. In the present case, the more pronounced structure observed with mitochondrial data could also underline the fact that our microsatellite loci were not as discriminating as they were expected to be [22]. Our study therefore highlighted a strong limitation of identifying population structure based on a single genetic marker and the obvious advantages of using combined molecular approaches. The additional use of genes supposedly under selection, *e.g.* SNPs (single nucleotide polymorphisms), or scan genomics approaches and detection of outlier loci [67], may be more relevant for future examination of swordfish - or more generally to large pelagic fish - population identification [68]. Detailed information about reproductive strategies, spawning areas and population dynamics in different areas within the IO are also needed to determine if the genetic homogeneity coincides with a demographical connectivity or only with evolutionary connectivity [69]. These general recommendation may be more pronounced when studies have concrete conservation implications such as fishery management of overexploited stocks by implementing Management Units and dedicated management measures.

### Interoceanic Isolation

Analyses of mitochondrial ND2 sequences and microsatellite polymorphism indicated significant isolation between oceans. Both molecular markers showed a significant level of genetic variance associated when comparing samples by Oceans, with a level of differentiation of the IO higher with the Atlantic Ocean (AO) than the Pacific Ocean (PO).

Indo-Pacific swordfish was until now considered to belong to a unique stock [15], [16], [53]. The fact that the IO was sampled more intensively than for previous studies and the use of the ND2 mitochondrial marker [22] could explain why this differentiation between Indian and Pacific samples was not detected in previous studies. Even if most of the water in the PO is circulated within the Pacific itself [70], some enters the Indonesia Seaway creating the Indonesian Throughflow current (ITF) flowing westward into the IO, supplying, to a large part, the global westward South Equatorial Current [56]. That current pattern therefore potentially transports swordfish larvae and juveniles from the important known spawning ground of western tropical Pacific [71], and could in turn be homogenizing the Indian and Pacific swordfish populations and justifying a unique Indo-Pacific population [15], [16], [20]. The absence in the PO and Indonesia of the haplotype #4 (very frequent in Atlantic, present at 5% in the IO) associated with the higher proportion of haplotype #21 in PO and Indonesia than in the rest of the IO (Figure 4) and the consequent high Ф_ST_ values observed between PO sample sets and the Indian sample sets are elements that strongly indicated an Indo-Pacific differentiation. Our results also showed that a significant part of genetic variance is associated with the Indo-Pacific differentiation (as well as low but still significant values of Ф_ST_ and F_ST_). One of the criteria previously used to discuss the interoceanic differentiation and consider the swordfish from Indo-Pacific as one population was the shared absence of Clade II in both oceans [53]. In our study, Clade II was not observed in the PO and Indonesia but was observed in the IO (at the low frequency of 2% but in all the IO areas); this could be viewed again as an argument against a unique Indo-Pacific population. A mark-recapture study around Australia also suggested this interoceanic disruption as the swordfish were recaptured in the ocean they have been released [54]. Therefore it should be more appropriate to consider the Indian and Pacific swordfish as belonging to separate stocks.

**Figure 5:**
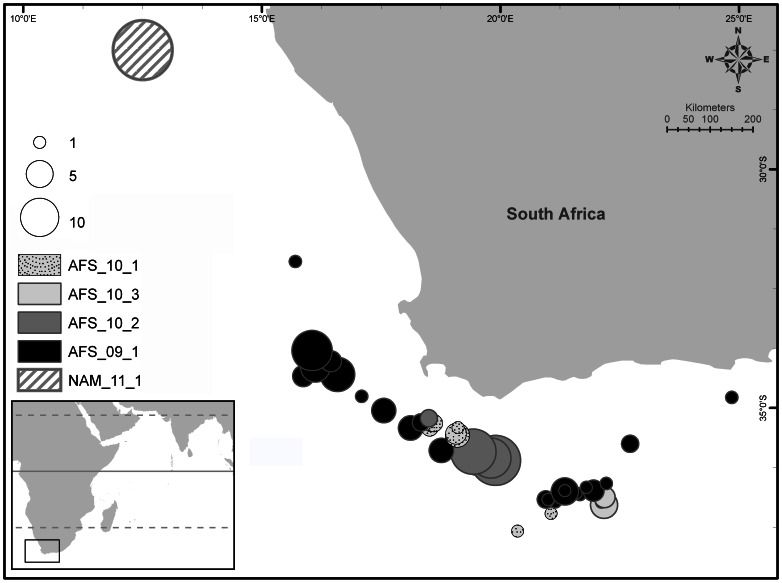
Sampling around South Africa and Namibia. Exact sampling size and location of the South Africa (AFS) and Namibia (NAM) sampling sets (see table 1 for details).

The high frequency of the haplotype #4 in the Namibia area (NAM; 30%) that decreased to less than 5% in the IO and absent in the PO and the consequent high Ф_ST_ values observed between NAM and Indian sample sets are elements that strongly indicated an Indo-Atlantic differentiation. Such differentiation observed is consistent with previous studies [15], [53] as well as with phylogeographic pattern reported for the bigeye tuna *Thunnus obesus* [72], the sailfish *Istiophorus platypterus,* and the blue marlin *Makaira nigricans* [73]. The Clade I was supposed to originate in the Pacific and the Clade II in the Atlantic. The co-occurrence of these two clades previously observed only in the Atlantic could be explained by unidirectional gene flow from the Indo-Pacific into the South Atlantic [55]. The fact that Clade II was now observed in the IO (at the low frequency of 2% but in all the IO areas) tends therefore to indicate that a flux of Atlantic swordfish into the IO could also occur. Such dispersal events from the Atlantic into the Indian Oceans were observed in only few species, because it necessitated strong swimming capacities to go against the Agulhas Current; it is the case of the hammerhead shark *Sphyrna lewini* [74], the green turtle *Chelonia mydas* [75] or the leatherback turtle *Dermochelys coriacea* [76], all being active swimmers in most of their life stages. The Agulhas Current [77] is one of the strongest currents in the world [78], with large westward current rings pinching off and transcending into the Atlantic, where it is also created the Agulhas Current Retroflexion (ACR) that flows back into the IO [77]. The greater warm and saline Agulhas system influences temperature and salinity of the AO over the full depth of the water column [79], creating an important gradient of temperature/salinity in short distance (up to 6° in less than 20 km; [78)]), and therefore being an environmental front in different pelagic habitat characteristics. Based on the fact that this front is highly variable in space and time [80], we suspect that the variability observed in mitochondrial signature of AFS samples (see Figure 5 for detailed localisation) could be attributed to specific pelagic habitats respectively used by Indian and Atlantic swordfish around South Africa, rather than an ontogenetic migration of individuals from the AO to the IO. The four sample sets from South Africa indeed showed contrasted mitochondrial signature, even if no significant genetic difference could be identified among them (p>0.05 for both Ф_ST_ and F_ST_). The sample sets AFS_09_1 (July - November 2009) and AFS_10_2 (April 2010) showed a mtDNA signature more related to the AO population while the two others sets (AFS_10_1 and AFS_10_3) were more similar to IO genetic signature (see details in Appendix F4a in File S1 and S5; congruently with a frequency of the haplotype #4 varying between 0 and 10%). This indicates that the boundary between Atlantic and Indian swordfish populations is not so strict and might be more considered as a transition zone between 17° and 23° east that is spatio-temporarily driven by the Agulhas Current activity. This last point engendered great perspectives in terms of management as the Atlantic stock is managed by the International Commission for the Conservation of Atlantic Tunas and the Indian stock by the Indian Ocean Tuna Commission, both legally being separated by the 20° east meridian. As already suggested for the bigeye tuna [71], [81], it might be very interesting to investigate the patterns of habitat use of IO and AO of large pelagic fishes in the South African waters, with a special focus on the sex-biased dispersal.

## Supporting Information

File S1 containing: Appendix F1: Geographic location of sampling areas and of the biogeographic provinces (Longhurst, 1998). Appendix F2: Swordfish sampling schedule. Appendix F3: Mismatch distribution for swordfish based on 1007 bp of the ND2 sequences. Appendix F4: Neighbor-joining trees showing the relationship between sample sets on the basis of pairwise genetic distances. Appendix S1: ND2 sequences pairwise ФST and D Jost’s values. Appendix S2: Microsatellites pairwise FST and D Jost’s values.
(DOCX)

## References

[1] Nakamura I (1985) FAO Species Catalogue. 5. Billfishes of the World. An Annotated and Illustrated Catalogue of Marlins, Sailfishes, Spearfishes and Swordfishes Known to Date. FAO Fisheries Synopsis 125: 65.

[2] Theisen TC, Bowen BW, Lanier W, Baldwin JD (2008) High connectivity on a global scale in the pelagic wahoo, *Acanthocybium solandri* (tuna family Scombridae). Molecular Ecology 17: 4233–4247. 19378403 10.1111/j.1365-294x.2008.03913.x

[3] Carlsson J, McDowell J, Carlsson J, Graves J (2007) Genetic Identity of YOY Bluefin Tuna from the Eastern and Western Atlantic Spawning Areas. Journal of Heredity 98: 23–28. 17158466 10.1093/jhered/esl046

[4] Gonzalez EG, Beerli P, Zardoya R (2008) Genetic structuring and migration patterns of Atlantic bigeye tuna, *Thunnus obesus* (Lowe, 1839). BMC Evol Biol 8: 252. 18798987 10.1186/1471-2148-8-252 PMC2559848

[5] Viñas J, Alvarado BremerJR, Pla C (2004) Inter-oceanic genetic differentiation among albacore (*Thunnus alalunga*) populations. Marine Biology 145: 225–232.

[6] Buonaccorsi VP, McDowell JR, Graves JE (2001) Reconciling patterns of inter-ocean molecular variance from four classes of molecular markers in blue marlin (*Makaira nigricans*). Molecular Ecology 10: 1179–1196. 11380876 10.1046/j.1365-294x.2001.01270.x

[7] Graves JE, McDowell JR (2006) Genetic analysis of white marlin (*Tetrapturus albidus*) stock structure. Bulletin of Marine Science 79: 469–482.

[8] Reeb CA, Arcangeli L, Block BA (2000) Structure and migration corridors in Pacific populations of the swordfish *Xiphias gladius*, as inferred through analysis of the mitochondrial DNA. Marine Biology 136: 1123–1131.

[9] Chow S, Clarke S, Nakadate M, Okazaki M (2007) Boundary between the north and south Atlantic populations of the swordfish (*Xiphias gladius*) inferred by a single nucleotide polymorphism at calmodulin gene intron. Marine Biology 152 (1): 87–93.

[10] Kotoulas G, Magoulas A, Tsimenides N, Zouros E (1995) Marked mitochondrial DNA differences between Mediterranean and Atlantic populations of the swordfish, *Xiphias gladius* . Molecular Ecology 4: 473–481.

[11] Garcia A, Mattiucci S, Damiano S, Santos MN, Nascetti G (2011) Metazoan parasites of swordfish, *Xiphias gladius* (Pisces: Xiphiidae) from the Atlantic Ocean: implications for host stock identification. ICES Journal of Marine Science: Journal du Conseil 68: 175–182.

[12] García-Cortés B, Mejuto J, Quintans M (2003) Summary of swordfish (*Xiphias gladius*) recaptures carried out by the spanish surface longline fleet in the Atlantic Ocean: 1984–2002. Col Vol Sci Pap ICCAT 55: 1476–1484.

[13] Sedberry G, Loefer J (2001) Satellite telemetry tracking of swordfish, *Xiphias gladius*, off the eastern United States. Marine Biology 139: 355–360.

[14] Neilson JD, Smith SC, Royer F, Paul SD, Porter JM, et al.. (2009) Investigations of Horizontal Movements of Atlantic Swordfish Using Pop-up Satellite Archival Tags. Tagging and Tracking of Marine Animals with Electronic Devices Reviews: Methods and Technologies in Fish Biology and Fisheries: Springer Netherlands. 145–159.

[15] Kadagi NI, Harris T, Conway N (2011) East Africa billfish Conservation and Research: Marlin, Sailfish and Swordfish Mark-Recapture field studies. 12 p.

[16] Chow S, Takeyama H (2000) Nuclear and mitochondrial DNA analyses reveal four genetically separated breeding units of the swordfish. Journal of Fish Biology 56: 1087–1098.

[17] Lu CP, Chen CA, Hui CF, Tzeng TD, Yeh SY (2006) Population genetic structure of the swordfish, *Xiphias gladius* (Linnaeus, 1758), in the Indian Ocean and West Pacific inferred from the complete DNA sequence of the mitochondrial control region. Zoological Studies 45: 269–279.

[18] Carey F, Robinson B (1981) Daily patterns in the activities of swordfish, *Xiphias gladius*, observed by acoustic telemetry. Fisheries Bulletin 79: 277–292.

[19] Takahashi M, Okamura H, Yokawa K, Okazaki M (2003) Swimming behaviour and migration of a swordfish recorded by an archival tag. Mar Freshwater Res 54: 527–534.

[20] Alvarado Bremer J, Mejuto J, Gomez-Marquez J, Boan F, Carpintero P, et al (2005) Hierarchical analyses of genetic variation of samples from breeding and feeding grounds confirm the genetic partitioning of northwest Atlantic and South Atlantic populations of swordfish (*Xiphias gladius* L.). Journal of Experimental Marine Biology and Ecology 327: 167–182.

[21] Jean C, Bourjea J, Jouen E, Taquet M (2006) Stock structure of the swordfish (*Xiphias gladius*) in the Southwest Indian Ocean: a preliminary study. Bulletin of Marine Science 79: 521–526.

[22] Bradman HM, Grewe PM, Appleton B (2011) Direct comparison of mitochondrial markers for the analysis of swordfish stock structure. Fisheries Research 109: 95–99.

[23] Muths D, Grewe P, Jean C, Bourjea J (2009) Genetic population structure of the Swordfish (*Xiphias gladius*) in the southwest Indian Ocean: Sex-biased differentiation, congruency between markers and its incidence in a way of stock assessment. Fisheries Research 97: 263–269.

[24] Poisson F, Fauvel C (2009) Reproductive dynamics of swordfish (*Xiphias gladius*) in the southwestern Indian Ocean (Reunion Island). Part 1: oocyte development, sexual maturity and spawning. Aquatic Living Resources 22: 45–58.

[25] Mejuto J, García-Cortés B, Ramos-Cartelle A (2006) An overview of research activities on swordfish (*Xiphias gladius*) and the bycatch species, caught by the Spanish longline fleet in the Indian Ocean.

[26] Yabe H, Ueyanagi S, Kikawa S, Watanabe H (1959) Study on the life history of the swordfish (*Xiphias gladius*) Report of the Nankai Regional Fisheries Research Laboratory. 10: 107–150.

[27] Francis R, Hixon M, Clarke M, Murawski S, Ralston S (2007) Ten commandments for ecosystem-based fisheries scientists. Fisheries 32: 217–233.

[28] IOTC–WPB10 (2012) Report of the Tenth Session of the IOTC Working Party on Billfish. Cape Town, South Africa. 66 p.

[29] IOTC (2011) Report of the Ninth Session of the IOTC Working Party on Billfishes. Seychelles, 4–8 July 2011: IOTC (Indian Ocean Tuna Commission). 63 p.

[30] IOTC (2011) IOTC–SC14 2011. Report of the fiftenth session of the IOTC scientific comity. Mahé, Seychelles: IOTC (Indian Ocean Tuna Commission). 272 p.

[31] Bradman HM, Muths D, Bourjea J, Grewe PM, Appleton B (2010) Characterisation of 22 polymorphic microsatellite loci in the broadbill swordfish, *Xiphias gladius* . Conservation Genetic Ressources 3: 263.

[32] Reeb CA, Arcangeli L, Block BA (2003) Development of 11 microsatellite loci for population studies in the swordfish, *Xiphias gladius* (Teleostei: Scombridae). Molecular Ecology Notes 3: 147–169.

[33] International Hydrographic Organization (1953) Limits of oceans and seas; publication IS, editor. Monaco.

[34] McCarthy C (1997) CHROMAS, Version 1.41. Brisbane: Griffith University.

[35] Thompson JD, Higgins DG, Gibson TJ (1994) CLUSTAL W: improving the sensitivity of progressive multiple sequence alignment through sequence weighting, positions-specific gap penalties and weight matrix choice. Nucleic Acids Research 22: 4673–4680. 7984417 10.1093/nar/22.22.4673 PMC308517

[36] Hall TA (1999) BIOEDIT: a user-friendly biological sequence alignment editor and analysis program for Windows 95/98/NT. Nucleic Acid Symposium Series 41: 95–98.

[37] Fu YX (1997) Statistical tests of neutrality of mutations against population growth, hitchhiking and background selection. Genetics 147: 915–925. 9335623 10.1093/genetics/147.2.915 PMC1208208

[38] Librado P, Rozas J (2009) DNASP v5: A software for comprehensive analysis of DNA polymorphism data. Bioinformatics 25: 1451–1452. 19346325 10.1093/bioinformatics/btp187

[39] Tamura K, Peterson D, Peterson N, Stecher G, Nei M, et al (2011) MEGA5: Molecular Evolutionary Genetics Analysis Using Maximum Likelihood, Evolutionary Distance, and Maximum Parsimony Methods. Molecular Biology and Evolution 28: 2731–2739. 21546353 10.1093/molbev/msr121 PMC3203626

[40] Nei M (1987) Molecular Evolutionary Genetics. New York: Columbia University Press.

[41] Excoffier L, Lischer H (2010) ARLEQUIN suite ver 3.5: A new series of programs to perform population genetics analyses under Linux and Windows. Molecular Ecology Resources 10: 564–567. 21565059 10.1111/j.1755-0998.2010.02847.x

[42] R Development Core Team (2010) R: A Language and Environment for Statistical Computing. Vienna, Austria: R Foundation for Statistical Computing.

[43] Wright F (1969) Volume 2: The theory of gene frequencies. Evolution and the genetics of population. Chicago: Chicago Press 512 p.

[44] Weir BS, Cockerham CC (1984) Estimating F-statistics for the analysis of population structure. Evolution 38: 1358–1370. 28563791 10.1111/j.1558-5646.1984.tb05657.x

[45] Van Oosterhout C, Hutchinson W, Wills D, Shipley P (2004) MICRO-CHECKER: software for identifying and correcting genotyping errors in microsatellite data. Mol. Ecol. Notes. Molecular Ecology Notes 4: 535–538.

[46] Pritchard JK, Stephens M, Donnelly P (2000) Inference of population structure using multilocus genotype data. Genetics 155: 945–959. 10835412 10.1093/genetics/155.2.945 PMC1461096

[47] Narum S (2006) Beyond Bonferroni: Less conservative analyses for conservation genetics. Conservation Genetics 7: 783–787.

[48] Benjamini Y, Yekutieli Y (2005) False discovery rate controlling confidence intervals for selected parameters. Journal of the American Statistical Association 100: 71–80.

[49] Jost L (2008) GST and its relatives do not measure differentiation. Molecular Ecology 17: 4015–4026. 19238703 10.1111/j.1365-294x.2008.03887.x

[50] Crawford NG (2009) SMOGD: software for the measurement of genetic diversity. Molecular Ecology Resources 10: 556–557. 21565057 10.1111/j.1755-0998.2009.02801.x

[51] Rousset F, Raymond M (1997) Statistical analyses of population genetic data: new tools, old concepts. Trends in Ecology & Evolution 12: 313–317. 21238087 10.1016/S0169-5347(97)01104-X

[52] Dupanloup I, Schneider S, Excoffier L (2002) A simulated annealing approach to define the genetic structure of populations. Molecular Ecology 11: 2571–2581. 12453240 10.1046/j.1365-294x.2002.01650.x

[53] Alvarado-Bremer JR, Mejuto J, Gomez-Marquez J, Boan F, Carpintero P, et al (2005) Hierarchical analyses of genetic variation samples from breeding and feeding grounds confirm the genetic partitioning of northwest Atlantic and South Atlantic populations of swordfish (*Xiphias gladius* L.). Journal of Experimental Marine Biology and Ecology 327: 167–182.

[54] Stanley C (2006) Determining the nature and extent of swordfish movement and migration in the eastern and western AFZ through an industry-based tagging program. CSIRO. 24 p.

[55] Palko B, Beardslay G, Richards W (1981) Synopsis of the biology of the swordfish, Xiphias gladius Linnaues. NOAA Technical Report NMFS Circular 441 FAO Fisheries synopsis, 2–15.

[56] Schott F, Xi S, McCreary P (2009) Indian Ocean circulation and climate variability. Reviews of Geophysics 47: RG1002.

[57] Longhurst A (1998) Ecological Geography of the Sea. San Diego, USA: Academic Press. 398 p.

[58] Bakun A, Roy C, Lluch-Cota S (1998) Coastal upwelling and other processes regulating ecosystem productivity and fish production in the western Indian Ocean. In: Okemwa E, Ntiba M, Sherman K, editors. Large Marine Ecosystems of the Indian Ocean Assessment, Sustainability, and Management. Malden: Blackwell Science. pp.103–141.

[59] Heileman S, Eghtesadi-Araghi P, Mistafa N (2009) Arabian Sea : LME. In: Sherman KaH, G (Editors), editor. The Unep large marine ecosystems report, a perspective on changing conditions in MLEs of the world’s regional seas. Nairobi, Kenya.

[60] Codispoti LA (1991) Primary productivity and carbon and nitrogen cycling in the Arabian Sea. In: Woods Hole Oceanographic Institution WH, U S., editor. US-JGOFS : Arabian Sea Process Study, U S Joint Global Ocean Flux Study Planning Report 13.

[61] Lévy M, Shankar D, André JM, Shenoi SC, Durand F, et al (2007) Basin-wide seasonal evolution of the Indian Ocean’s phytoplankton blooms. Journal of geophysical research 112: 14.

[62] Ménard F, Potier P, Jaquemet S, Romanov E, Sabatié R, Cherel Y (2012) Pelagic cephalopods in the western Indian Ocean : New information from diets of top predators. Deep-Sea Research II.

[63] Ménard F, Labrune C, Shin YJ, Asine AS, Bard FX (2006) Opportunistic predation in tuna: a size based approach. Marine Ecology Progress Series 323: 223–231.

[64] Potier M, Marsac F, Cherel Y, Lucas V, Sabatié R, et al (2007) Forage fauna in the diet of three large pelagic fishes (lancetfish, swordfish and yellowfin tuna) in the western equatorial Indian Ocean. Fisheries Research 83: 60–72.

[65] Prugnolle F, de Meeus T (2002) Inferring sex-biased dispersal from population genetic tools: a review. Heredity 88: 161–165. 11920116 10.1038/sj.hdy.6800060

[66] Hoey JJ (2986) A review of sex ratio by size data for western North Atlantic swordfish; 1986; Miami. NMFS, SEFC. 21.

[67] Nielsen EE, Hemmer-Hansen J, Larsen PF, Bekkevold D (2009) Population genomics of marine fishes: identifying adaptive variation in space and time. Molecular Ecology 18: 3128–3150. 19627488 10.1111/j.1365-294X.2009.04272.x

[68] Russello MA, Kirk SL, Frazer KK, Askey PJ (2012) Detection of outlier loci and their utility for fisheries management. Evolutionary Applications 5: 39–52. 25568028 10.1111/j.1752-4571.2011.00206.x PMC3353332

[69] Waples RS, Gaggiotti O (2006) What is a population? An empirical evaluation of some genetic methods for identifying the number of gene pools and their degree of connectivity. Molecular Ecology, 15, 1419–1439. 10.1111/j.1365-294X.2006.02890.x 16629801

[70] Lukas R, Yamagata T, McCreary JP (1996) Pacific low-latitude western boundary currents and the Indonesian throughflow. Journal of Geophysical Research 101: 12209–12216.

[71] Nishikawa Y, Honma M, Ueyanagi S, Kikawa S (1985) Average Distribution of Larvae of Oceanic Species of Scombrid Fishes, 1956–81. S Series Far Seas Fishery Research Laboratory, Shimizu 12: 99.

[72] Chow S, Okamoto H, Miyabe N, Hiramatsu K, Barut N (2000) Genetic divergence between Atlantic and Indo-Pacific stocks of bigeye tuna (*Thunnus obesus*) and admixture around South Africa. Molecular Ecology 9: 221–227. 10672166 10.1046/j.1365-294x.2000.00851.x

[73] Graves JE, McDowell JR (2003) Stock structure of the world’s istiophorid billfishes: a genetic perspective. Marine and Freshwater Research 54: 287–298.

[74] Duncan KM, Martin AP, Bowen BW, De Couet HG (2006) Global phylogeography of the scalloped hammerhead shark (*Sphyrna lewini*). Molecular Ecology 15: 2239–2251. 16780437 10.1111/j.1365-294X.2006.02933.x

[75] Bourjea J, Lapegue S, Gagnevin L, Broderick D, Mortimer JA, et al (2007) Phylogeography of the green turtle, *Chelonia mydas*, in the Southwest Indian Ocean. Molecular Ecology 16: 175–186. 17181729 10.1111/j.1365-294X.2006.03122.x

[76] Luschi P, Lutjeharms JRE, Lambardi P, Mencacci R, Hughes GR, et al (2006) A review of migratory behaviour of sea turtles off southeastern Africa. South African Journal of Science 102: 51–58.

[77] Richardson PL, Lutjeharms JRE, Boebel O (2003) Introduction to the “Inter-ocean exchange around southern Africa”. Deep-sea res II 50: 1–12.

[78] Lutjeharms JRE (2007) Three decades of research on the greater Agulhas Current Ocean Science. 3: 129–147.

[79] Beal L, De Ruijter W, Biastoch A, Ranier Z, 136 SWIG (2011) On the role of the Agulhas system in ocean circulation and climate. Nature 472: 429–436. 21525925 10.1038/nature09983

[80] Lutjeharms JRE (2006) The Agulhas Current; Springer, editor. Verlag, Heidelberg. 329 p.

[81] Durand JD, Collet A, Chow S, Guinand B, Borsa P (2005) Nuclear and mitochondrial DNA markers indicate unidirectional gene flow of Indo-Pacific to Atlantic bigeye tuna (*Thunnus obesus*) populations, and their admixture off southern Africa. Marine Biology 147: 313–322.

