# Supplementary material for: Multi-Genetic Marker Approach and Spatio-Temporal Analysis Suggest There Is a Single Panmictic Population of Swordfish Xiphias gladius in the Indian Ocean
Source: PLoS One. 2013 May 22;8(5):e63558. doi: 10.1371/journal.pone.0063558 (PMC3661515; doi:10.1371/journal.pone.0063558)
Supplement: File S1 — containing: Appendix F1: Geographic location of sampling areas and of the biogeographic provinces (Longhurst, 1998). Appendix F2: Swordfish sampling schedule. Appendix F3: Mismatch distribution for swordfish based on 1007 bp of the ND2 sequences. Appendix F4: Neighbor-joining trees showing the relationship between sample sets on the basis of pairwise genetic distances. Appendix S1: ND2 sequences pairwise ФST and D Jost’s values. Appendix S2: Microsatellites pairwise FST and D Jost’s values. (DOCX) [file pone.0063558.s001.docx]

**Appendix F1:** Geographic location of identified sampling areas and associated area name (in bold) used for data spatial and temporal analyses (see also Table 1). Grey colours differentiate the areas one to each other. Lines indicate the biogeographic Longhurst provinces ans associated names in regular (from Longhurst, 1998).


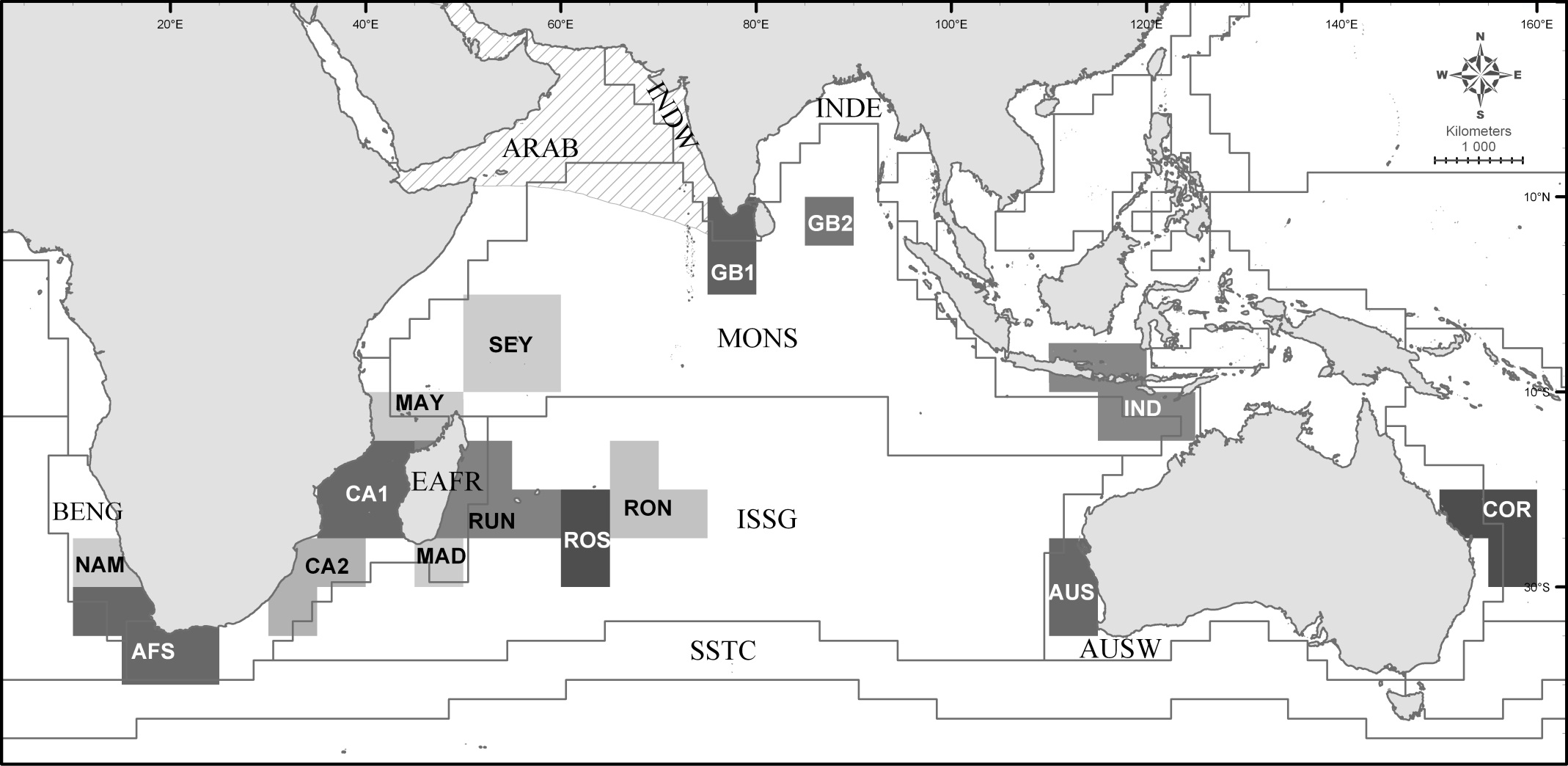


**Appendix F2:** Swordfish sampling schedule. The grey areas are related to the targeted seasons, the black squares indicate when sampling occurred per area (see Table 1 for area names).


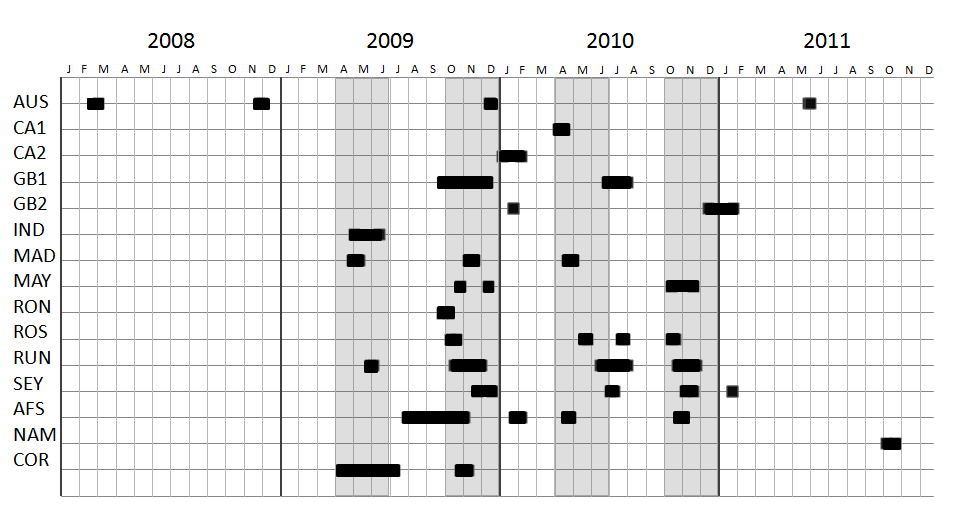


**Appendix F3.** Mismatch distribution for swordfish based on 1007bp of the ND2 sequences. The black line represents the observed and the dashed grey line the simulated pairwise differences, respectively.

**
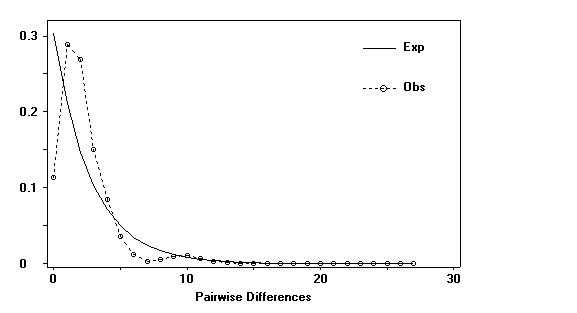
**


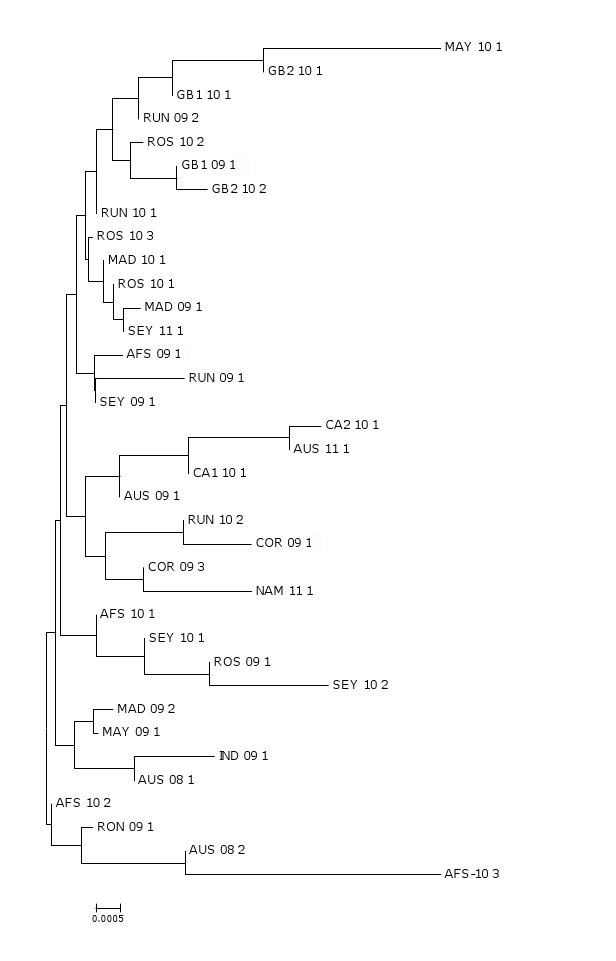
**Appendix F4.** Neighbor-joining trees showing the relationship between sample sets on the basis of pairwise genetic distances estimated with (3a) ND2 sequences and (3b) microsatellite datasets**.**
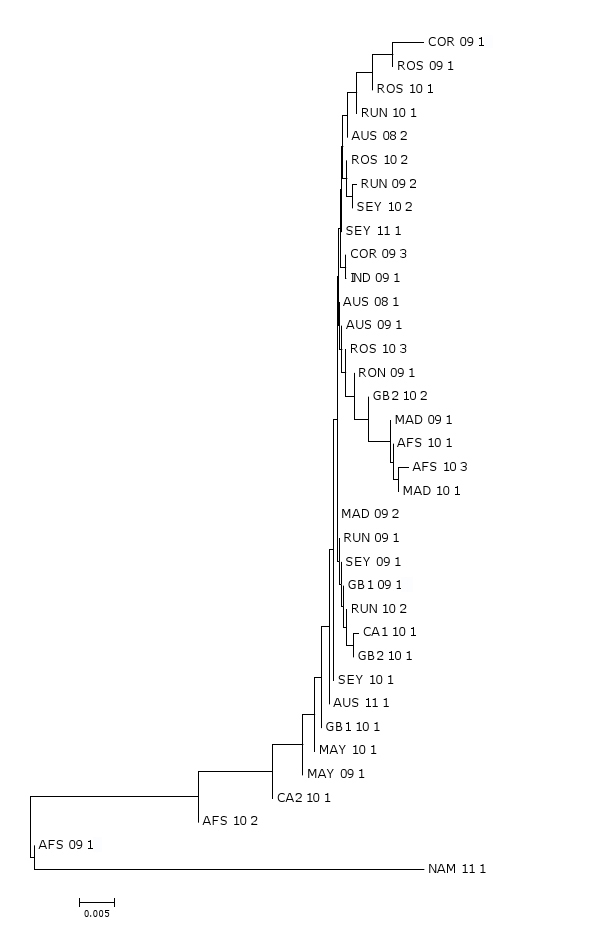


4a.

4b.

**Appendix S2:** ND2 sequences pairwise ФST values for swordfish sampling sets are shown in below diagonal (p value < 0.05 are indicated in bold characters, P value = 0.000 are indicated by *). D Jost’s value for swordfish sampling sets are shown in above diagonal (values above 0.1 are indicated in bold).

**Appendix S3:** Microsatellites Pairwise FST values for swordfish sampling sets are shown in below diagonal (p value < 0.05 are indicated in bold characters, p value = 0.000 are indicated by *; D Jost’s value for swordfish sampling sets are shown in above diagonal (values above 0.01 are indicated in bold).
